# Augmentation of single tooth extraction socket with deficient buccal walls using bovine xenograft with platelet-rich fibrin membrane

**DOI:** 10.1186/s12903-023-03554-2

**Published:** 2023-11-17

**Authors:** Riham Mohamed Elbanna, Medhat Sameh Abdelaziz, Hesham Ebrahim Alameldeen

**Affiliations:** 1 Faculty of dentistry, Beni-Suef University, Beni-Suef, Egypt; 2 Faculty of Oral and Dental Medicine, Prosthodontics department, Future University in Egypt, Cairo, Egypt; 3 Faculty of Oral and Dental Medicine, Future University in Egypt, Cairo, Egypt

**Keywords:** Alveolar ridge preservation, Alveolar ridge augmentation, Platelet-rich fibrin, Platelet concentrates, Bone grafts, CBCT, Radiographic changes, Tooth extraction

## Abstract

**Background:**

Different techniques and materials such as bone grafts and bioactive agents have been used for alveolar ridge augmentation in extraction sockets with a defective wall, there is not a specific material or technique that has resulted in superior outcomes or prevented total bone loss.

**Objectives:**

This clinical study aims to evaluate radiographically the effectiveness of using bovine xenograft with platelet-rich fibrin (PRF) membrane on vertical and horizontal alveolar ridge dimensional changes following tooth extraction that are complicated by buccal bone loss.

**Materials and methods:**

This study was conducted in Egypt on fourteen patients with a single posterior tooth indicated for extraction. A preoperative cone-beam computed tomography (CBCT) scan confirmed more than 50% loss in buccal bone in each tooth. Extraction sockets were packed with minced PRF clots mixed with a bovine xenograft. Each extraction socket was sealed by PRF membranes. CBCT scans, performed before tooth extraction and after 6 months, were used to assess alveolar ridge changes both vertically and horizontally.

**Results:**

There was a significant gain in the buccal and middle of the extraction socket bone height, recording 86.01% (6.33 mm) and 206.45% (9.6 mm), respectively. There was an insignificant bone loss in the lingual bone height and width, recording − 8.49% (-1.06 mm) and − 13.39% (1.05 mm), respectively. The results also showed a non-significant decrease in alveolar bone density (-14.06%) between pre-operative bone present apical to the extraction socket and newly formed bone inside the socket.

**Conclusions:**

Ridge preservation/augmentation techniques using a bone graft mixed with PRF and covered by PRF membranes in fresh extraction sockets complicated by the loss of buccal bone result in buccal bone augmentation and a reduction in horizontal and vertical ridge collapse after tooth extraction.

**Clinical relevance:**

The bovine xenograft in conjunction with PRF can be used immediately after extraction for ridge preservation, providing adequate bone width and height for implant placement.

## Introduction

Alveolar ridge bone quantity and quality are important factors for osseointegration and the long-term survival of dental implants [1, 2]. Many clinical studies reported a loss of 11–22% of alveolar bone height and 29–63% of alveolar bone width after tooth extraction, which occurred mainly on the buccal side [3–10]. Bone loss happens at a fast rate during the first 3–6 months post-extraction, then it slows down to 0.5–1% bone loss per year [11, 12].

There are different techniques for socket preservation depending on the type of bone grafts used, such as autografts, allografts, xenografts, and alloplasts, in addition to bioactive agents and the use of membranes to achieve the concept of guided bone regeneration (GBR). [13–16] The introduction of socket seal techniques resulted in more effective outcomes in comparison to spontaneous healing or using biomaterials without a seal [17–21], The socket seal utilizes different materials, such as soft tissue grafts or collagen matrix [12, 17, 22].

The use of bone grafts with socket seal techniques, or GBR, is beneficial in preventing soft tissue graft or membrane collapse into the socket area and also in enhancing new bone formation by acting as a scaffold maintaining a space for bone ingrowth and blood vessel formation [22–24]. Growth factors delivered in fresh extraction sockets enhance bone regeneration either used alone or in combination with other materials. Platelet-rich fibrin (PRF) is a second generation of platelet concentrates, which consists of platelets that release different growth factors, including platelet-derived growth factor, transforming growth factor, vascular endothelial growth factor, insulin-like growth factor, basic fibroblast growth factor, and epidermal growth factor [25–27]. The PRF clot provides a strong fibrin matrix that protects growth factors from proteolysis and acts as a scaffold for carrying cells essential for tissue regeneration. In addition to the advantage that PRF is completely autologous and cost-effective [28–30].

Although different techniques and materials have been used for alveolar ridge preservation/augmentation, no specific material or technique has resulted in superior outcomes or prevented total bone loss [31]. Thus, the present study aimed to investigate the effectiveness of using bovine xenograft with platelet-rich fibrin membrane for augmentation of the alveolar ridge following tooth extraction. The null hypothesis is that there is no difference in bone width and height after using bovine xenograft with platelet-rich fibrin membrane in freshly extracted sockets.

## Materials and methods

The present study was conducted according to the Declaration of Helsinki for the ethical principles of medical research involving human subjects and was approved by the research ethics committee of the Faculty of Dentistry, Beni-Suef University (#REC-FDBSU/05012023-03/ER). The study was registered on clinicaltrials.gov with registration number NCT05791123. First registration 30/03/2023.Fourteen patients, who required implant treatment after posterior tooth extraction were selected from the outpatient clinic of the Department of Prosthodontics at the Future University Faculty of Oral and Dental Medicine (FUE) and the Beni-Suef University Hospital for dental treatment.

### Inclusion criteria

- Patients requiring implant treatment after tooth extraction due to either un restorable caries lesions or affected periodontium.
- Age group ranging from 20 to 60 years old.
- Patients who are able to tolerate the procedure under local anesthesia.
- The socket wall is characterized by loss of buccal bone (> 50%) confirmed by preoperative cone beam computed tomography (CBCT) and presence of other socket walls; mesial, distal and lingual walls.

### Exclusion criteria

- Diabetic patients that are not well controlled.
- Patients with hypertension, hepatic and renal disorders.
- Systemic diseases concerned with bone metabolism.
- heavy smokers were also excluded from the study.

### Study power and sample size calculation

Sample size calculation was done according to a study [32], that attempted alveolar bone preservation after tooth extraction using different materials. The study reported a true difference in the mean ± SD of bone change by approximately 5.71 ± 3.45. According to the previous study, we deduced that the minimum proper sample size was 7 extraction sites to achieve 80% power at α level = 0.05. To compensate for a drop-out rate, the number was increased to 14 extraction sites. The sample size was calculated using (PS: Power and Sample Size Calculation 3.1) software.

### Surgical protocol

**Fig. 1:**
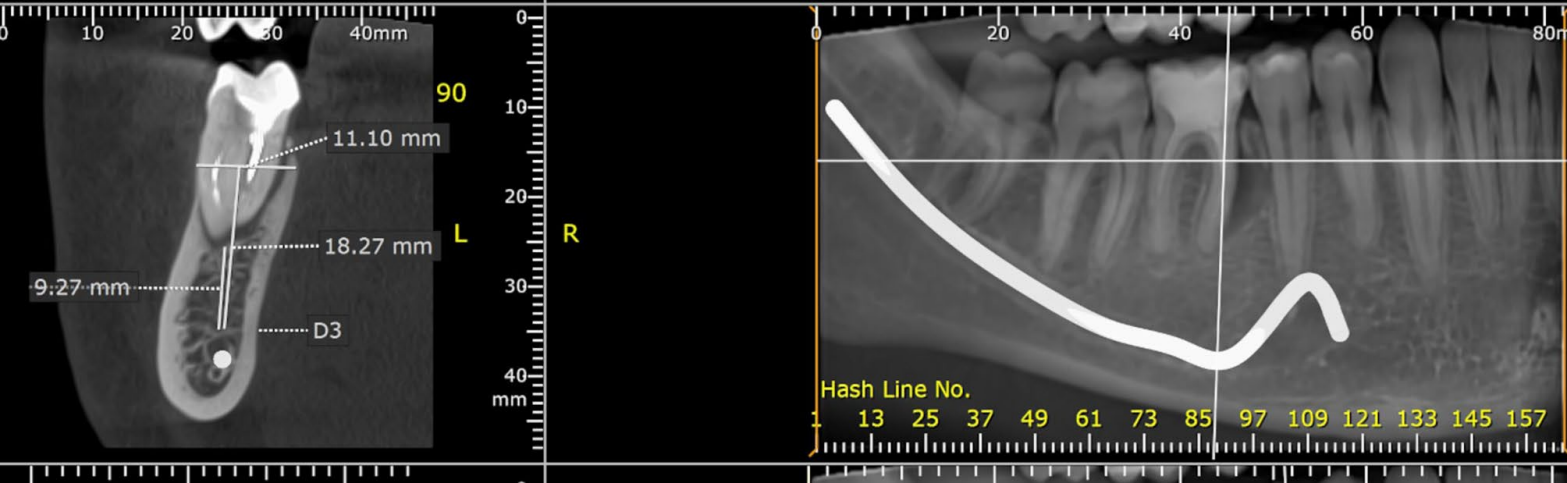
Preoperative CBCT for non-restorable lower left first molar. Note the absence of buccal bone

Preoperative radiographic assessment using cone beam computed tomography (CBCT) (PaX-i3D Green; VATECH)with 90 kV and 12 mA for 12 s as exposure parameters and with the recommended field of view by the manufacture (Fig. 1) was used to visualize the status of the existing alveolar bone in all patients. A full mucoperiosteal flap was raised buccally to expose the bone crest. Atraumatic tooth extraction protocol was used to perform all teeth extractions, which included sectioning of multirooted teeth, the use of periotomes to luxate the roots, the preservation of bony walls of the socket (mesial, distal, lingual, and inter-radicular walls), and the avoidance of applying any pressure to bony walls after tooth extraction (socket squeeze). Extraction sockets were irrigated and curetted.

### Platelet-rich fibrin (PRF) preparation

**Fig. 2:**
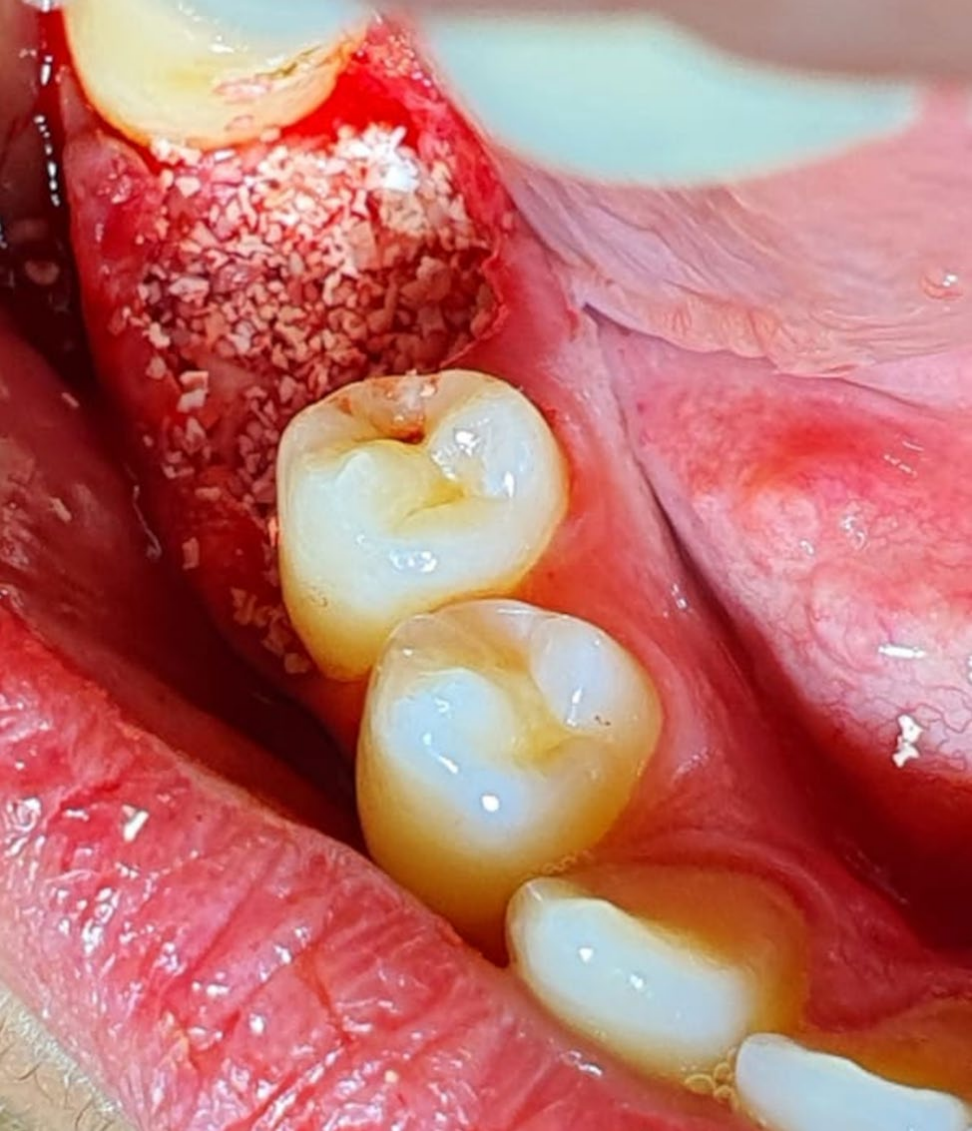
Clinical photograph showing an extraction socket filled with bone graft mixed with minced PRF.

**Fig. 3:**
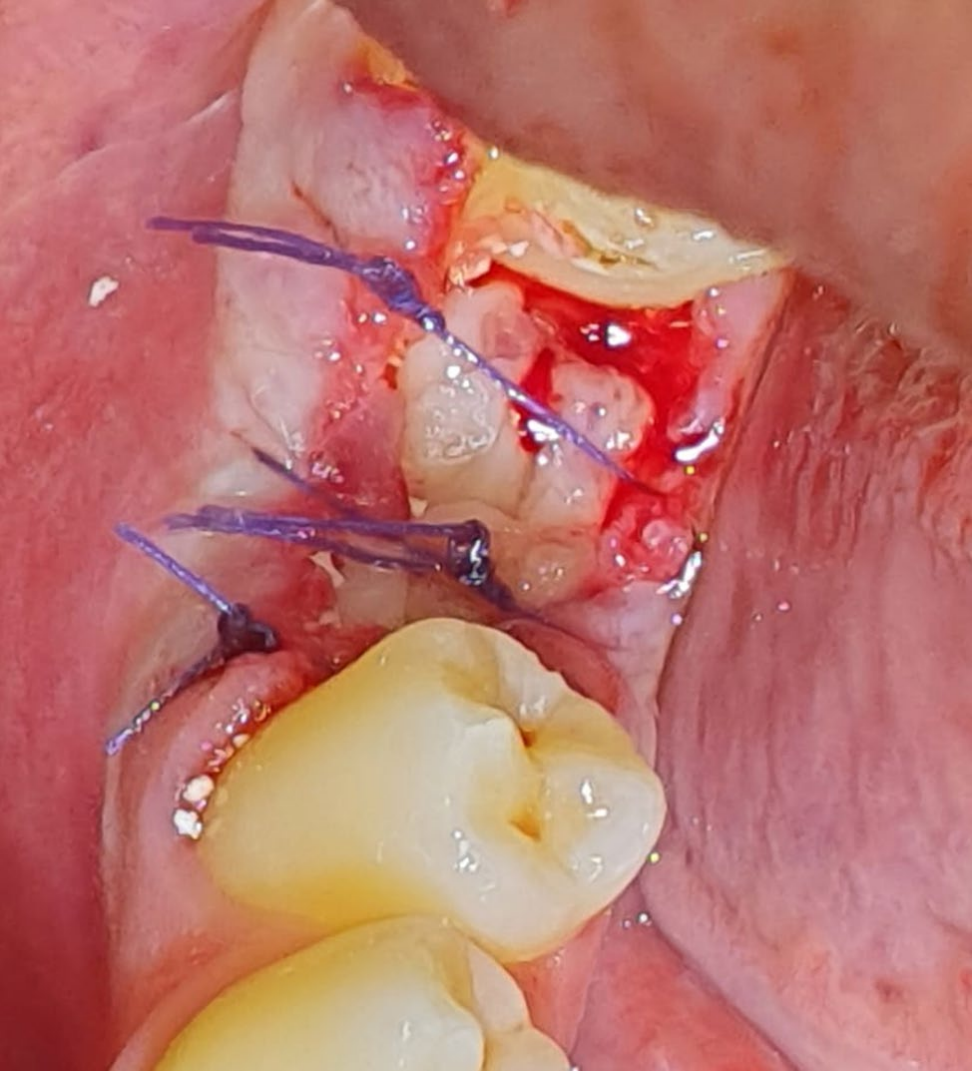
Clinical photograph showing an extraction socket sealed with multiple PRF membranes and sutured

Intravenous blood was withdrawn just prior to surgery and placed in six sterile vacutainer tubes of 5 mL capacity without anticoagulant and immediately centrifuged at 3,000 revolutions per minute (rpm) for 10 min (LABFISH solutions GMBH, Germany) After blood centrifugation, a structured fibrin clot is formed in the middle of the tube, with red blood cells at the bottom and platelet***-***poor plasma (PPP) at the top. Only the PRF clot was easily taken from the tubes and separated using a sterile tweezer and scissors. A small red blood cell layer was preserved after the removal of PPP to keep “the buffy coat” layer rich in white blood cells (WBCs) within the clot. Minced PRF was achieved by cutting one or more PRF clots into small pieces using a scissor and then mixing it with the bovine xenograft(Inno Oss B, Cowellmedi ; Korea) and placing it inside the extraction socket (Fig. 2) to build the lost buccal wall and fill the socket. Other PRF clots were used as membranes. The PRF membranes were prepared by squeezing the PRF clot between two sterile glass slabs to squeeze out the fluids in the fibrin clot. Multiple PRF membranes were then placed to seal the extraction socket and cover the underlying bone graft buccally and occlusally. The flap was positioned in its original position with simple interrupted sutures using 4 − 0 absorbable polyglycolic acid sutures (Fig. 3).

### Post-surgical phase

Postoperative oral analgesics (Brufen 400 mg tablets) on need and systemic antibiotics (Amoxicillin 500 mg t.d.s.) were prescribed for 7 days to avoid postsurgical infection. A mouth rinse of 0.12% chlorhexidine gluconate, 3 times daily for 4 weeks, was prescribed for plaque control. The sutures were removed after two weeks. The postoperative evaluation after 6 months was achieved by CBCT, and dental implants are placed at the healed sites.

### Radiographic analysis

**Fig. 4:**
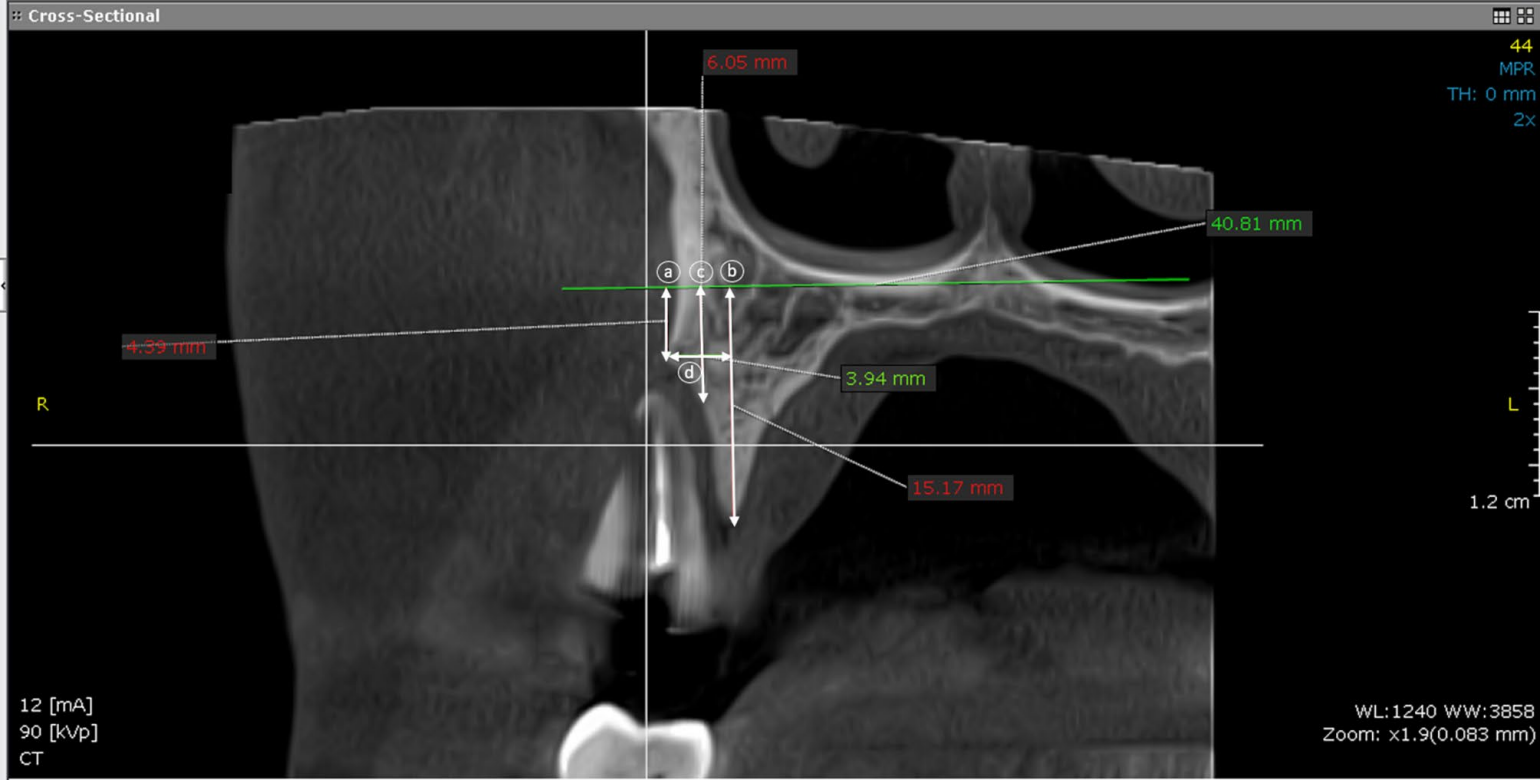
Radiographic measurement of bone width and height before surgery. **(a)** The measurement from the floor of the maxillary sinus to the top of the buccal alveolar bone **(b)** The measurement from the floor of the maxillary sinus to the top of the palatal alveolar bone **(c)** The measure from the floor of the maxillary sinus to the socket wall halfway between the palatal and buccal bony peaks. **(d)**The bone width is achieved by measuring the distance from points **(a)** to **(b)**

**Fig. 5:**
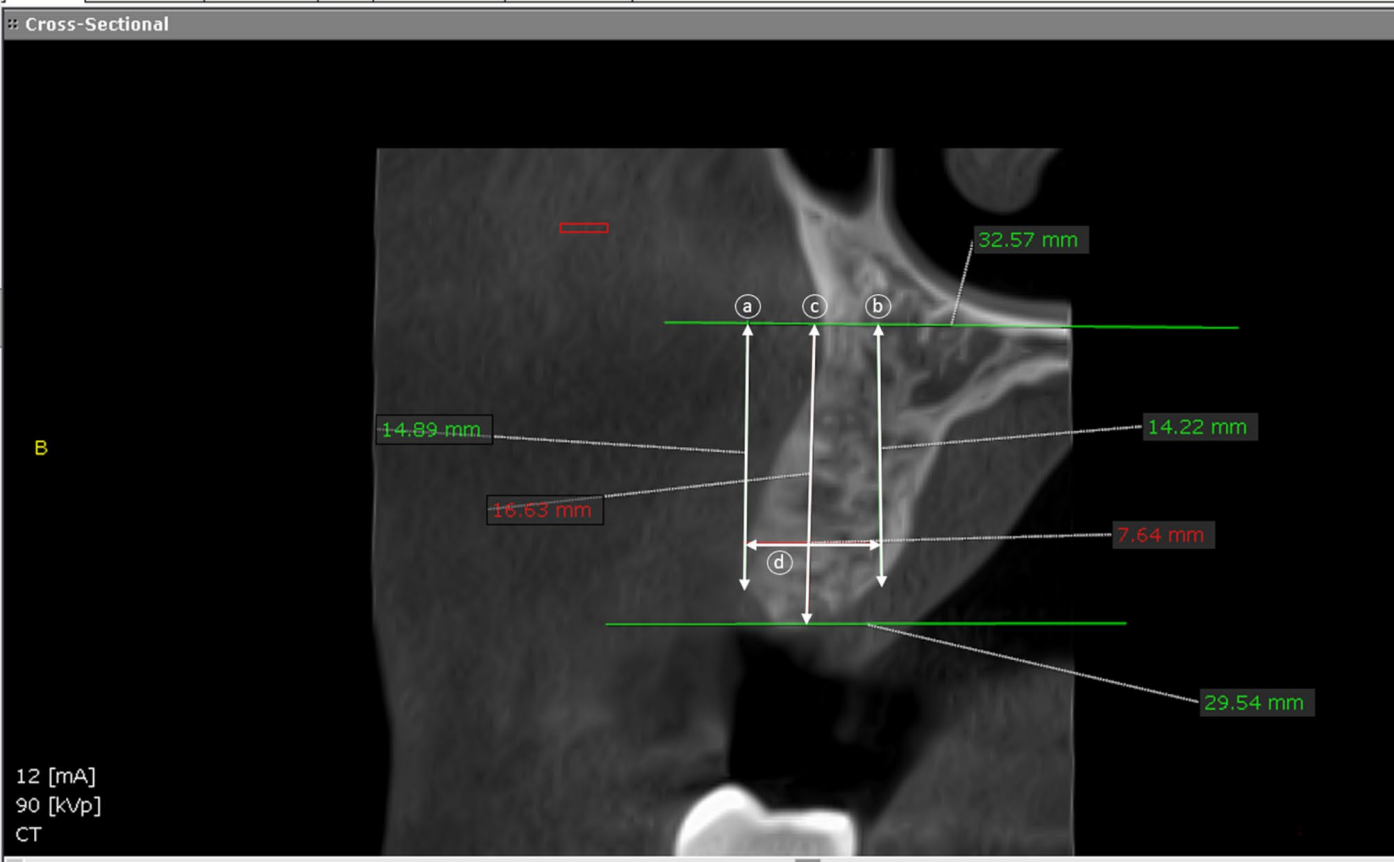
Radiographic measurement of bone width and height 6 months after surgery. **(a)** The measurement from the floor of the maxillary sinus to the top of the buccal alveolar bone **(b)** The measurement from the floor of the maxillary sinus to the top of the palatal alveolar bone **(c)** The measure from the floor of the maxillary sinus to the halfway between the palatal and buccal plates of bone. **(d)** The bone width is achieved by measuring the distance from points **(a)** to **(b)**

CBCT was obtained at baseline (before tooth extraction) and at 6 months postoperatively. The CBCT scans was analyzed using Blue Sky software program. (Blue Sky V4.11.2 64bit, IL, USA). The area of interest was selected using the software program (x, y) coordination tool [33–35]. Linear radiographic measurements in mm between 2 marked points were made on the CBCT radiographs [34, 35] (Figs. 4 and 5). Bone changes after tooth extraction were measured by assessing the width and height of the alveolar bone. Three lines were drawn to divide the socket:

- Buccal alveolar bone height: The distance from the top of the buccal alveolar bone to the bottom of the mandible.
- Lingual alveolar bone height: The distance from the top of the lingual alveolar bone to the bottom of the mandible.
- The middle of the socket: The distance from the socket wall halfway between the lingual and buccal bony peaks to the bottom of the mandible.
- The alveolar width: The distance between the drawn lines (a) and (b).

**Fig. 6:**
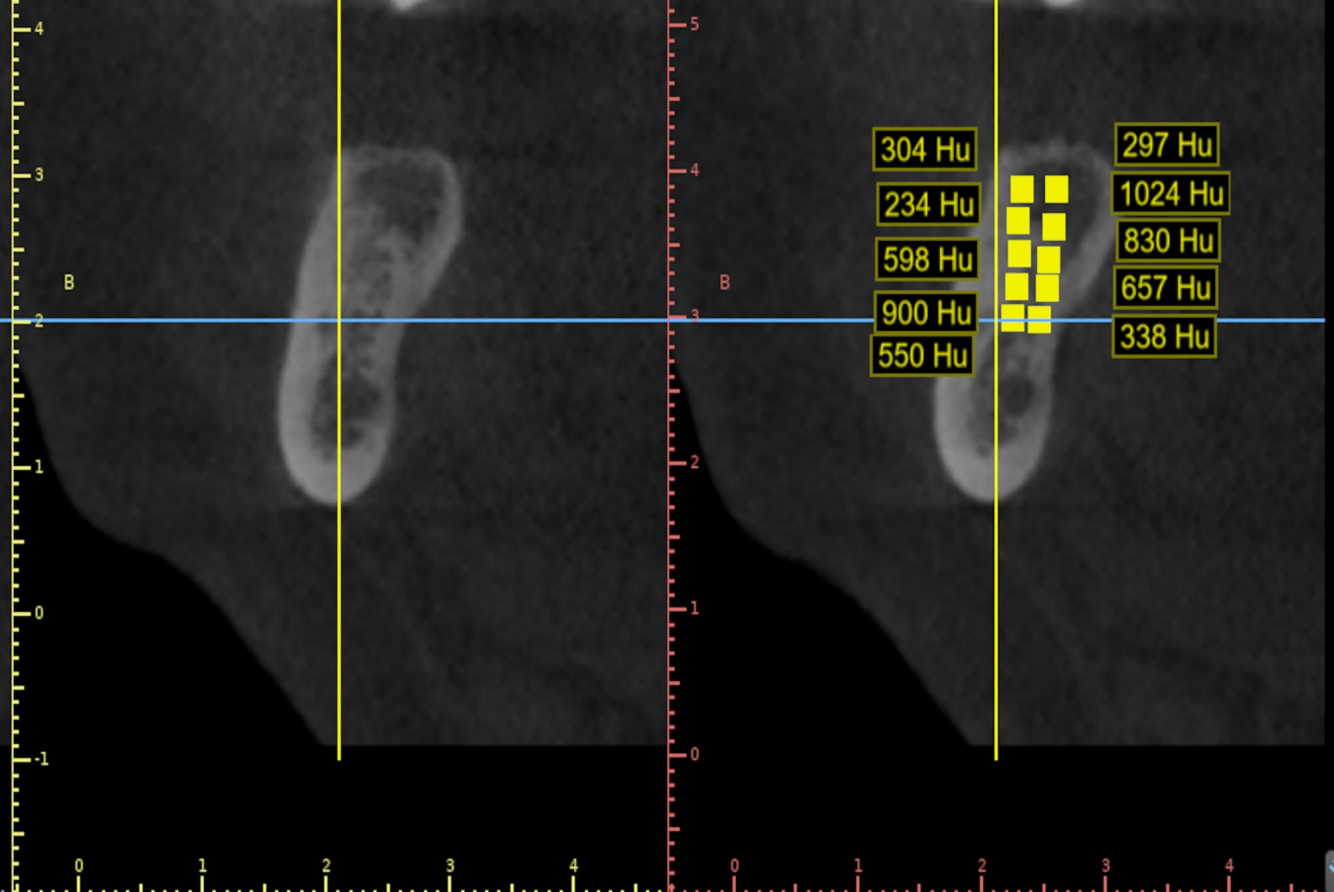
Measurement of the bone density in Hounsfield units

In the case of maxillary sites, the floor of the maxillary sinus was taken as a reference line instead of the bottom of the mandible. To locate the healed extraction site mesiodistally on CBCT, a measured distance was achieved between the adjacent teeth and the middle position of the tooth on CBCT taken before extraction. Then, the same records were obtained on the post-operative CBCT.

#### Bone density

The bone density was calculated using the Hounsfield units (HU) which were automatically calculated using the blue sky software program built-in density measuring tool [33, 36]. The surface area of the defect was divided into 10 successive parallel points then the Gray level of each point was calculated, and the mean value of these points was recorded [33, 36]. This represents the bone density at the defect site. These measurements were taken at the baseline and after 6 months, and then the results were compared to check for any change in bone density. (Fig. 6).

### Statistical methodology

Data were statistically evaluated using the IBM SPSS software package, version 24.0. (Armonk, NY: IBM Corp.). Data were presented as means and standard deviations for each parameter and compared at different time intervals using a paired t-test for dependent variables.

## Results

14 patients with mean age 40.3 ± 11.2 years completed this study (9 females and 5 males). The distribution of extraction sites is shown in Table 1.

Regarding the buccal alveolar bone height, there was a significant increase of (86.01%) after six months, while the lingual alveolar bone height, revealed an insignificant decrease by (8.49%). (Table 2)

**Table 1:** Tooth Extraction Sites Distribution

	Premolars	Molars	P-value
Upper	77.8% (5)	22.2% (2)	**0.617**
Lower	75% (4)	25% (3)	

*P; Probability Level at P ≤ 0.05. NS; Insignificant Different using Chi-Square Test*

**Table 2:** Alveolar Bone Height, Width and Density CBCT Measurements among Six Months Postoperative

	Buccal Alveolar Bone Height	Lingual Alveolar Bone Height	Middle of the Socket	Alveolar Bone Width	Alveolar Bone Density
Preoperative (Baseline)	7.36 ± 5.37	12.49 ± 5.09	4.65 ± 3.89	7.84 ± 3.11	902.20 ± 163.39
Postoperative (After Six Months)	13.69 ± 6.50	11.43 ± 5.16	14.25 ± 6.86	6.79 ± 2.47	775.33 ± 247.61
P-value	0.00*	**0.07**	0.04*	**0.066**	**0.749**

M ± SD; Mean ± Standard Deviation, P; Probability Level at P ≤ 0.05. *; Significant Different using Paired T-test for dependent values

For the middle of the socket, there was a significant increase of (206.45%) after six months recording a P-value < 0.05 using a paired t-test. On the other hand, alveolar bone width and density revealed an insignificant ant decrease by (13.39%) and (14.06%) respectively, with a P-value > 0.05. (Table 2)

## Discussion

Ridge preservation is defined as alveolar preservation within the bony envelope existing after tooth extraction, while ridge augmentation is defined as increasing the volume of bone beyond the bony envelope at the time of extraction [37] Clinically, it is often difficult to differentiate between those two techniques, and in the literature, they are described mainly as “extraction socket preservation.“ [14].

A systematic review done by Juodzbalys G et al. in 2019 [14] presented an extraction socket classification based on morphological characteristics and set up a decision tree for ridge preservation/augmentation after extraction procedures. Ridge preservation/augmentation was recommended in the following cases; when there are facial soft tissue deficiencies so aesthetic results are jeopardized, the presence of buccal wall defect > 50%, and horizontal bone loss ˃ 2 mm. Also, functional reasons were discussed, such as when implant primary stability is difficult to achieve because the existing bone apical to the extraction socket is less than 3 mm, damage of inter-radicular bone, or an absence of contact between the implant and bony walls. Moreover, risk-related factors were mentioned, such as the risk of considerable bone loss (due to thin buccal wall thickness post-operatively < 2 mm, multiple extractions, and a thin periodontal biotype < 1 mm), the risk of maxillary sinus or nasal floor perforation, and the risk of post-operative infection development due to the presence of bony lesions ˃ 5 mm [14].

The extent of bone loss after tooth extraction depends on different factors, such as the thickness of facial bone and its existence [38], tooth angulation [39],the presence of periodontal or endodontic infection, the number of adjacent teeth needed to be extracted, and traumatic tooth extraction practices that resulted in bony wall fracture [40, 41]. During healing, if there is no sufficient bony wall barrier, this could result in fibrous tissue ingrowths into the fresh extraction socket, impairing bone regeneration and causing considerable loss of alveolar bone [42].

Regarding facial bone thickness, a clinical study evaluated 39 patients by CBCT and showed that thin periodontal biotypes with facial bone thickness ≤ 1 mm experienced gradual bone loss after 2 months of healing, extending to 62% of the bone height. While a thick periodontal biotype with a facial bone thickness of more than 1 mm revealed a less obvious bone loss, reaching only 9% of bone height [38].

While concerning the number of adjacent extraction teeth, an experimental study [43] revealed that buccal bone obtains its blood supply from adjacent teeth, and they classified extraction sockets as single or multiple extractions. Also, Al-Shabeeb et al. [44] proved that neighboring tooth extraction resulted in advanced bone loss. Moreover, extensive bone loss was presented when multiple adjacent immediate implants were done.

Various treatment protocols were recommended to decrease dimensional changes that occur post-extraction and/or to renew damaged socket walls: (1) Atraumatic tooth extraction (low trauma tooth extraction technique), (2) Flapless tooth extraction concept, and (3) Ridge preservation/augmentation techniques. Atraumatic tooth extraction included the use of special extraction instruments and devices in addition to multi-rooted teeth sectioning in order to decrease pressure on the facial and inter-radicular bones and remove the root parts separately [45].

Regarding flap elevation, it was known that flap elevation has a negative effect on bone remodeling during healing due to the disruption of the periosteal blood vessels and the increase in postoperative local inflammation. However, a systematic review proved that bone loss with or without flap elevation had no differences observed after a healing period of 6 months [46]. Conversely, a flapless procedure should be recommended whenever possible [24].

Studies that compared different ridge preservation/augmentation procedures with spontaneous healing after tooth extraction demonstrated a greater orofacial dimension of alveolar bone after healing when ridge preservation/augmentation procedures were adopted. While spontaneous healing resulted in massive bone alterations in many cases, which risked the possibility of the dental implant as a prosthetic treatment [46, 47].

In the present study, a significant gain in the buccal bone height was achieved that reached 86.01% (6.33 mm), and the socket filled with new bone that was 206.45% (9.6 mm) in the middle of the socket. An insignificant bone loss occurred in the lingual alveolar bone width and height which was − 8.49% (-1.06 mm) and − 13.39% (-1.05 mm) respectively. This was in accordance with a randomized, controlled clinical trial that applied various materials to preserve alveolar bone after tooth extraction. The alveolar bone loss was 1.3 mm in bone width and 0.57 mm in bone height after 3 months with the use of a xenograft. While in sites without socket preservation techniques, the horizontal and vertical bone loss were 2.79 and 1.74 mm, respectively, at 3 months post-surgically [48]. In another study, [32] extraction sites were filled with a composite material of hydroxyapatite/collagen for ridge preservation. After 3 months post-surgery, the results showed that the middle of the socket floor was elevated by 5.71 ± 3.45 mm, while the bone width was decreased by 1.02 ± 1.64 mm, and the height was decreased at the lingual side by 0.35 ± 1.73 mm.

Moreover, an animal study done on beagle dogs observed the sequence of healing processes in extraction sockets with buccal bone- deficient defects. In the spontaneous healing of deficient sites, large dimensional shrinkage (81.85 ± 6.60%) occurred. While in ridge augmentation sites, the final dimensions (104.74 ± 6.18%) were comparable to those of the pristine alveolar ridge, and this was attributed to the space provided by biomaterials placed in ridge augmentation sites, into which new bone formed continuouslys [49]. For this reason, a review article [24] recommended that in the case of severe buccal bone loss (> 50%), especially in the aesthetic zone, preservation of hard tissue using bone substitute material before implant placement is adopted.

Although, there was insignificant decrease in the bone density in the present study, this could be attributed to the newly formed bone is cancellous bone with more air spaces in comparison to the socket walls which may cause decrease in the bone density as seen in (Fig. 6).

Regarding the benefits of combining PRF with bone grafts, a study done by Thakkar DJ et al. [50] compared the use of PRF with DFDBA bone graft and the use of DFDBA alone for socket preservation. After 6 months, the loss of hard tissue width and height in the PRF + DFDBA group was − 0.75 (0.493) mm and − 1.083 (0.429) mm, respectively. While in the group of DFDBA alone, the reduction values of ridge width and height were − 1.361 (0.703) mm and − 1.389 (0.502) mm respectively, which proved that the incorporation of PRF into the bone graft resulted in reduced bone loss.

In the present study, the use of PRF membrane gave the advantage of sealing the socket to cover the bone graft without the need to coronally advance the flap or to move the keratinized tissue palatally/lingually. Also, PRF is well known to accelerate soft tissue healing and promote angiogenesis [51], which resulted in faster soft tissue closure for the socket.

One of the limitations of this study is during CBCT data aquesition,it is difficult to reproduce the same multi planar view on pre- and postoperative cbct reconstructions due to the the different degree of the patient mouth opening.which may cause minor errors in the measurements of the outcome. Also the build-up, image acquisition of CBCT scanners may cause artefacts for grey level measurements in bone quality assessment, how ever the CBCT offers a measurment device with less radaiation biohazards in comparison to the medical CT devices.

## Conclusions

The present study proved that the placement of a bone graft mixed with PRF and covered by PRF membranes in a fresh extraction socket that was complicated by the loss of buccal bone resulted in buccal bone augmentation and reduced horizontal and vertical ridge collapse after tooth extraction.

## Data Availability

The datasets used and/or analysed during the current study available from the corresponding author on reasonable request.
